# Deletion of Wild-type p53 Facilitates Bone Metastatic Function by Blocking the AIP4 Mediated Ligand-Induced Degradation of CXCR4

**DOI:** 10.3389/fphar.2021.792293

**Published:** 2022-02-01

**Authors:** Qiji Li, Min Wang, Leli Zeng, Wei Guo, Yuandong Xu, Chenxin Li, Yingrong Lai, Liping Ye, Xinsheng Peng

**Affiliations:** ^1^ Department of Orthopaedic Surgery, The Seventh Affiliated Hospital, Sun Yat-sen University, Shenzhen, China; ^2^ Guangdong Provincial Key Laboratory of Digestive Cancer Research, Guangzhou, China; ^3^ Department of Orthopaedic Surgery, The First Affiliated Hospital of Sun Yat-sen University, Guangzhou, China; ^4^ Scientific Research Center, The Seventh Affiliated Hospital, Sun Yat-sen University, Shenzhen, China; ^5^ Department of Urology, The Seventh Affiliated Hospital, Sun Yat-sen University, Shenzhen, China; ^6^ Department of Pathology, The First Affiliated Hospital of Sun Yat-sen University, Guangzhou, China

**Keywords:** wild-type p53, AIP4, CXCR4, prostate cancer, bone metastasis

## Abstract

**Background:** Management of patients with prostate cancer and bone metastatic disease remains a major clinical challenge. Loss or mutation of p53 has been identified to be involved in the tumor progression and metastasis. Nevertheless, direct evidence of a specific role for wild-type p53 (wt-p53) in bone metastasis and the mechanism by which this function is mediated in prostate cancer remain obscure.

**Methods:** The expression and protein levels of wt-53, AIP4, and CXCR4 in prostate cancer cells and clinical specimens were assessed by real-time PCR, immunohistochemistry and western blot analysis. The role of wt-p53 in suppressing aggressive and metastatic tumor phenotypes was assessed using *in vitro* transwell chemotaxis, wound healing, and competitive colocalization assays. Furthermore, whether p53 deletion facilitates prostate cancer bone-metastatic capacity was explored using an *in vivo* bone-metastatic model. The mechanistic model of wt-p53 in regulating gene expression was further explored by a luciferase reporter assay and chromatin immunoprecipitation (ChIP) assay.

**Results:** Our findings revealed that wt-p53 suppressed the prostate cancer cell migration rate, chemotaxis and attachment toward the osteoblasts *in vitro*. The bone-metastatic model showed that deletion of wt-p53 remarkably increased prostate cancer bone-metastatic capacity *in vivo*. Mechanistically, wt-p53 could induce the ligand-induced degradation of the chemokine receptor CXCR4 by transcriptionally upregulating the expression of ubiquitin ligase AIP4. Treatment with the CXCR4 inhibitor AMD3100 or transduction of the *AIP4* plasmid abrogated the pro-bone metastasis effects of *TP53* deletion.

**Conclusion:** Wt-p53 suppresses the metastasis of prostate cancer cells to bones by regulating the CXCR4/CXCL12 activity in the tumor cells/bone marrow microenvironment interactions. Our findings suggest that targeting the wt-p53/AIP4/CXCR4 axis might be a promising therapeutic strategy to manage prostate cancer bone metastasis.

## Introduction

Prostate cancer is currently the second most commonly diagnosed cancer and the fifth leading cause of cancer-associated death in men worldwide (Sung et al., 2021). At diagnosis, approximately 5% of patients have clinically distant metastasis in the United States (Siegel et al., 2018), and the incidence of metastatic disease is typically markedly increased among Asians (Ito, 2014). The most common site of distant metastasis occurs in bones with an incidence rate of 84% in advanced prostate cancer (Gandaglia et al., 2014). Furthermore, the survival of patients with bone metastatic prostate cancer is very poor with overall survival of only 3% after 5 years (Nørgaard et al., 2010). As the management of patients with bone metastatic disease remains a major clinical challenge, it is imperative that we elucidate its molecular mechanisms to develop novel therapeutic strategies for prostate cancer.

Increasing evidence supports a critical role for the interaction between disseminated cancer cells and the bone marrow microenvironment during tumor colonization and metastasis growth (Yoneda and Hiraga, 2005). The CXCR4/CXCL12 axis was originally described as an essential mediator of hematopoietic stem cell (HSC) homing to and retention within the bone marrow (Broxmeyer et al., 2005) and shown to be a crucial player in organ-specific dissemination (Kucia et al., 2005). Both bone marrow endothelial cells, as well as osteoblasts, express and secrete the CXCR4 ligand CXCL12 (also known as SDF-1) (Sun et al., 2005). The chemokine SDF-1 is abundant in the bone marrow where it attracts CXCR4 expressing cells (Dar et al., 2011). Interestingly, CXCR4 is found on the surface of prostatic tumors cells, and its expression progressively correlates with the malignant degree, peaking in bone metastasis specimens (Clarke et al., 2009). Disseminated tumor cells could hijack the same molecular mechanisms used by HSCs, and directly compete for occupancy of the endosteal osteoblastic niches during localization to the marrow (Müller et al., 2001; Shiozawa et al., 2011). Treatment with CXCR4 antagonists significantly reduces bone marrow colonization of metastatic cancer cells and growth of intraosseous metastasis (Sun et al., 2005; Festuccia et al., 2019). However, not much is currently known about the dysregulation of the chemokine receptor CXCR4 levels in prostate cancer progression.

p53 is well known to function as a tumor suppressor that triggers cell cycle arrest, apoptosis, or senescence in response to cellular stress signal, to preserve genomic stability (Kastenhuber and Lowe, 2017). Loss of wild-type p53 (wt-p53) function perturbs cellular and organismal homeostasis, and even contributes to tumor initiation and malignant progression (Park et al., 2018). Importantly, we and others have shown that wt-p53 exerts pleiotropic effects by regulating multiple steps of the metastatic cascade, including epithelial-mesenchymal transition (EMT), extracellular matrix interactions, stemness and anoikis, through the induction of downstream genes (Ren et al., 2013; Tang et al., 2020). Deletion and/or mutation of the *TP53* gene which encodes p53 is a common feature of human cancers (Hainaut et al., 1998). Significantly, a bioinformatics study revealed a stepwise increase of TP53 mutations toward prostate cancer aggressiveness, showing the lowest expression in benign prostatic hyperplasia (19.0%), followed by primary prostate cancer (26.2%), and finally, metastatic castration-resistant disease (53.3%) (Schlechte et al., 1998; Network, 2015; Robinson et al., 2015). Nevertheless, direct evidence for the specific role of wt-p53 in bone metastasis and the mechanism by which p53 regulates the bone metastatic propensity of tumor cells is required.

Here, we report that the deletion of wt-p53 results in a significant increase in bone metastasis formation in mouse models. Mechanistically, p53 transcriptionally activates the downstream ubiquitin ligase AIP4, and promotes degradation of the chemokine receptor CXCR4 to block the trafficking of CXCR4-positive prostate cancer cells to the bone marrow. Our findings indicate that targeting the wt-p53/AIP4/CXCR4 axis might be a promising therapeutic strategy to manage prostate cancer bone metastasis.

## Materials and Methods

### Cell Lines and Cell Culture

The human prostate cancer cell lines C4-2B and PC-3, along with the mouse pre-osteoblastic cell line MC3T3-E1 were obtained from the American Type Culture Collection (ATCC) and authenticated by using short tandem repeat (STR) profiling. C4-2B cells were grown in T-medium (GIBCO, Waltham, MA, USA) supplemented with 10% FBS, PC-3 cells in F-12K medium (GIBCO) supplemented with 10% FBS, and MC3T3-E1 cells in Alpha Minimum Essential Medium (GIBCO). All cell lines were grown in a humidified incubator at 37°C in 5% CO_2_ and were routinely tested for *mycoplasma* using the LookOut *Mycoplasma* PCR Detection Kit (Sigma-Aldrich, St. Louis, MO, USA).

### Western Blot Analysis

Western blotting was performed as described previously (Li et al., 2021). The following primary antibodies were used in this study: anti-p53 wild-type (clone PAb1620 (Puca et al., 2008); Merck Millipore, Billerica, MA, USA), anti-CXCR4 (ab124824; Abcam, Cambridge, United Kingdom), anti-AIP4 (NBP2-55083; Novus Biologicals, Littleton, CO, USA), anti-α-tubulin (T9026; Sigma-Aldrich), and anti-GAPDH (#97166; Cell Signaling Technology, Danvers, MA, USA) antibodies.

### Human Tissue Specimens

A total of 46 paraffin-embedded and archived prostate cancer specimens from 46 individual patients were collected for this study and histopathologically diagnosed. These tissues were obtained from primary tumors during surgery or needle biopsy at the First Affiliated Hospital of Sun Yat-sen University between January 2010 and December 2017. Clinical information on the samples is summarized in detail in Supplementary Table S1. Prior informed consent from the patients and ethics approval from the Institutional Research Ethics Committee were obtained for the use of clinical samples for research purposes. This study was conducted in accordance with the 1975 Declaration of Helsinki.

### Immunohistochemistry

IHC staining was performed to assess the protein levels of target genes in the 46 paraffin-embedded prostate cancer tissue section using the Histostain-Plus Kit (ThermoFisher, Waltham, MA, USA) following the manufacturer’s protocols, as described previously (Ye et al., 2019). The following primary antibodies were used in this study: anti-p53 wild-type (clone PAb1620 (Puca et al., 2008); Merck Millipore), anti-CXCR4 (ab124824; Abcam) and anti-AIP4 (NBP1-89180; Novus Biologicals) antibodies. From IHC analysis, positive cells were defined as any yellow or brown immunostained cells by antibodies according to the manufacturer’s instructions. The percentages of wt-p53, AIP4, and CXCR4 positive cells in specimens from patients with prostate cancer were counted by a Java operating open source image analysis program ImageJ (Vayrynen et al., 2012; Grishagin, 2015).

### Plasmid Construction, Transfection, and Establishment of Stable Cell Lines

Full-length cDNA encoding human *TP53* and *AIP4* was PCR-amplified and cloned into a pMSCV-puro-retro vector (Takara, Shiga, Japan). The sgRNAs against human TP53 in LentiCRISPRv2-puro, as well as the shRNAs against human CXCR4 and AIP4 in vector pLKO.1-puro, were purchased from Transheep Bio (Shanghai, China). The human AIP4 promoter sequences generated by PCR amplification were cloned into the pGL3 luciferase reporter plasmid (Promega, Madison, WI, USA) to construct the corresponding luciferase reporters. CRISPR/Cas9 systems/plasmids were stably transfected into the prostate cancer cells (1 × 10^5^/ml) using Lipofectamine 3,000 (Invitrogen, Waltham, MA, USA) according to the manufacturer’s instructions. After 2 weeks, positive clones were selected based on their resistance to puromycin (800 mg/ml; North China Pharmaceutical, Shijiazhuang, China), and analyzed by reverse transcription-polymerase chain reaction (RT-PCR). Cells infected with the pGL3 luciferase retrovirus were selected with 250 μg/ml G418.

### RNA Extraction, Reverse Transcription, and Quantitative Real-Time PCR

Total RNA was extracted from tissues and cultured cells with TRIzol reagent (Invitrogen) according to the manufacturer’s instructions, and then reverse-transcribed to cDNA by M-MLV Reverse Transcriptase (Promega). qRT-PCR analysis was carried out on a CFX96 Real-Time System C1000 Cycler (Bio-Rad Laboratories, Hercules, CA, USA) using TB Green Fast qPCR Mix (Takara). The mRNA expression data was normalized to the housekeeping gene glyceraldehyde-3-phosphate dehydrogenase (GAPDH). The relative expression levels of the target genes were calculated as 2^−[(Ct of gene) - (Ct of GAPDH)]^, where Ct represents the threshold cycle for each transcript. The sequences of primers for qRT-PCR are as follow: AIP4 forward 5′- AGC​GTA​GTC​AGC​TTC​AAG​GAG -3’; AIP4 reverse, 5′- AGG​TGG​CAA​TGG​ACC​AAG​AG -3’; GAPDH forward 5′- AAG​GTG​AAG​GTC​GGA​GTC​AA -3’; and GAPDH reverse, 5′- AAT​GAA​GGG​GTC​ATT​GAT​GG -3’.

### Luciferase Activity Assay

The luciferase activity assay was conducted as described previously (Li et al., 2017a). Prostate cancer cells (3 × 10^3^ cells/well) were plated in 48-well culture plates in triplicate. After 24 h culture, 100 ng of the indicated luciferase reporter constructs or the control luciferase plasmid, together with 3 ng pRL-TK Renilla plasmid (Promega) used as an internal control, was transfected into the tumor cells using Lipofectamine 3,000 (Invitrogen) according to the manufacturer’s instructions. Luciferase and Renilla signals were detected 48 h after transfection using the Dual-Luciferase Reporter Assay Kit (Promega) according to the manufacturer’s recommendations. The relative promoter activity was presented as the ratio of firefly to Renilla luciferase activity. The sequences of the *AIP4* promoter and the mutants driving the luciferase promoter have been listed as Supplementary Table S2. The putative or mutated p53-binding sequences in the indicated *AIP4* promoter regions cloned into the pGL3 luciferase reporter plasmid are as follows: wild-type: 5′-ACA​AGC​CCC​AGC​AGG-3’; mutant: 5′-GAG​GTA​AAA​GTA​GTT-3’.

### Chromatin Immunoprecipitation

The ChIP assay was carried out using the SimpleChIP Enzymatic Chromatin IP Kit (Magnetic Beads) (Cell Signaling Technology) according to the manufacturer’s protocol, as described previously (Li et al., 2021). Briefly, prostate cancer cells (4 × 10^6^) were cultured in a 100 mm culture dish and transfected with TP-p53 plasmids. Next, the cells were treated with 1% formalin to cross-link proteins to DNA. Thereafter, 1× glycine was added to terminate the cross-linking. Then, the indicated cells were lysed in SDS buffer, sonicated and incubated with 5 μg of anti-p53 antibody (sc-126; SANTA CRUZ, Santa Cruz, CA, USA) or anti-immunoglobulin G antibody (I8765; Sigma-Aldrich) overnight at 4°C with constant rotation. The protein–DNA complexes were enriched by adding ChIP-grade protein G magnetic beads and incubating for 2 h at 4°C with rotation. Then, the immunoprecipitated chromatin was rinsed with low- and high-salt ChIP buffer. After releasing DNA fragments from the protein/DNA cross-linking, PCR was performed using the primers: AIP4-promoter forward, 5′- AGA​CTA​AGA​TGG​CAG​CCG​GT -3’; and AIP4-promoter reverse, 5′- TCT​AGA​CCA​CTT​CCG​CGC​T -3’. The ChIP efficiency of certain binding sites was evaluated using the percentage of chipped DNA against input chromatin.

### Wound Healing Assays

The tumor cell migration ability was assessed by a wound-healing assay *in vitro*. Prostate cancer cells (2 × 10^4^) were seeded on 6-well plates and cultured in a medium containing 10% FBS until reaching approximately 90% confluence. The plated cells were wounded with a sterile 10-μL pipette tip. Then the culture medium was removed and replaced immediately with the conditioned medium generated from pre-osteoblast MC3T3-E1 cultures under 10% serum conditions. Images of the monolayer wound were observed under a phase-contrast microscope. The migratory ability of the prostate cancer cells was calculated as the ratio of the culture area after 24 h to the culture area at 0 h.

### Transwell Chemotaxis Assays

Chemotactic migration of prostate cancer cells toward a mimic bone microenvironment was performed using a 24-well chemotaxis chamber (Costar; CORNING, Corning, NY, USA). Briefly, pre-osteoblast MC3T3-E1 (2.5 × 10^4^ cells) cells were grown for 16 h in the lower chamber containing an 800 µL culture medium with 15% FBS. Prostate cancer cells (2 × 10^4^) were suspended in a serum-free medium and plated in the upper chambers. After 48 h incubation at 37°C, non-migrated cells were wiped off with a cotton swab, and the cells that had penetrated through the membrane to the lower surface were fixed with 4% paraformaldehyde and stained with hematoxylin, before being photographed and quantified by counting under a microscope. The migration and invasive ability of prostate cancer cells were calculated as the mean number of cells in five random high-magnification fields (×200).

### *In Vitro* Competitive Colocalization Assays

*In vitro* competitive colocalization assays were performed as described previously (Shiozawa et al., 2011). Murine pre-osteoblast MC3T3-E1 (1 × 10^3^ cells) cells were seeded evenly into 6-well plates supplemented with culture medium containing 10% FBS for 24 h, and then labeled with CellTracker Blue CMAC (Invitrogen) according to the manufacturer’s protocol. *TP53*/*TP53*-knockout (KO) and vector control prostate cancer cells (2 × 10^2^) labeled with CellTracker Red CMTPX (Invitrogen) and CellTracker Green CMFDA (Invitrogen), respectively, were added to the culture system together. After 2 and 24 h incubation at 37°C, respectively, the cells in the 6-well plates were observed using a confocal laser scanning microscope system (FV1000; Olympus Medical Systems, Tokyo, Japan).

### *In Vivo* Bone-Metastatic Model

Male BALB/c nude mice (Slac-Jingda Animal Laboratory, Hunan, China) were housed in a barrier facility under controlled lighting (12-h light/dark). Prostate cancer cells (5 × 10^5^) were suspended in 100 μL PBS solution and injected into the left ventricle of 4-6-weeks-old anesthetized mice by a 28.5-gauge needle attached to a 0.5 ml syringe. The luciferase bioluminescence in the mice injected with tumor cells was imaged and measured twice a week using the IVIS Spectrum imaging system (Caliper Life Sciences, Hopkinton, MA, USA). The growth curve for bone metastasis burden in the hind limbs was quantified based on the luminescent signal intensity. Mice were euthanized on the indicated day. The hind-leg bones were surgically harvested, fixed in 4% neutral-buffered formalin, decalcified with 10% EDTA for 3 weeks, and then paraffin-embedded for hematoxylin and eosin (H&E) staining. The metastatic nodules were observed visually and counted by histomorphometry using Image-Pro Plus v7.0 (Media Cybernetics, Rockville, MD, USA). The incidence and number of bone metastasis in the hind limbs were preliminarily evaluated by the luminescent signal in the mouse bones, and then validated by IHC and histomorphometry after the mice were euthanized. The tumor burden of each mouse was defined as the area of bone occupied by tumor cells validated by H&E staining.

### Statistical Analysis

Statistical analyses were carried out using the SPSS v21.0 software package (SPSS, Chicago, IL, USA). For comparisons of two groups, the Student’s t-test was used; the analysis of variance (ANOVA) test was applied to analyze the difference between more than two groups. Pearson’s correlation analysis was conducted to evaluate the correlation of protein levels with gene expression based on the IHC data of prostate cancer tissues. Survival curves were plotted with the Kaplan-Meier method and were compared by the log-rank test. Data were considered statistically significant for *p* < 0.05 in all cases.

## Results

### Depletion of Wt-p53 Promotes Bone Metastasis and Shortens Survival in Mice

**FIGURE 1:**
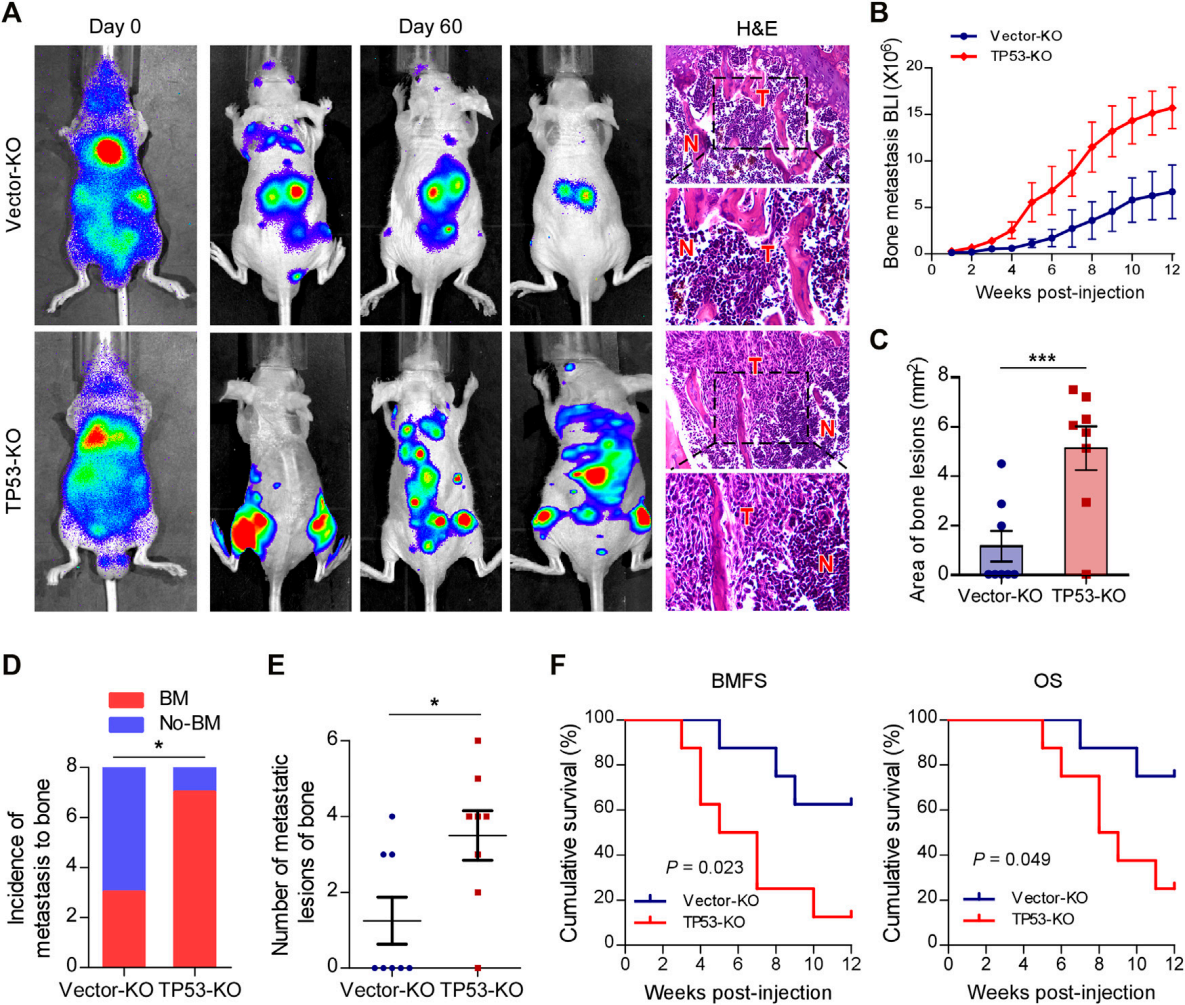
Depletion of wt-p53 promotes bone metastasis and shortens survival in mice. **(A)** Representative BLI and histological images (H&E) of bone lesions from mice inoculated with C4-2B/*TP53*-KO or C4-2B/Vector-KO cells (*n* = 8/group). Red arrows indicate bone lesions by tumor (T). N, adjacent non-tumor tissues. **(B)** Growth curve for bone metastasis burden as quantified by BLI in the indicated groups of C4-2B cells. **(C)** Histomorphometric quantification of tumor area in hind limbs from each experimental group. **(D)** Frequency of bone metastasis detected in each group (χ^2^ test). **(E)** Numbers of metastatic lesions in bones from each mouse in two groups. **(F)** Kaplan-Meier curves of bone metastasis-free survival (BMFS) and overall survival (OS) in mice from each experimental group (log-rank test). Error bars represent the means ± SEM. *p < 0.05, ***p < 0.001. *P* values were based on *t*-test unless otherwise indicated.

To investigate the role of wt-p53 in prostate cancer bone metastasis, we used the CRISPR/Cas9 technology to genetically ablate the endogenous *TP53* expression in C4-2B cells (Supplementary Figures S1A,B). Next, a rapid bone-metastatic model was established by intracardiac injections of luciferase-labeled vector or *TP53*-KO C4-2B cells in BALB/c-nude mice. Bioluminescence imaging (BLI) of C4-2B luciferase-labeled cells was used to track the tumor cells twice a week *in vivo* with the IVIS Spectrum imaging system. Figure 1A showed the BLI and H&E images of bone lesions from representative mice in each experimental group. Notably, the growth curve and histomorphometry showed that *TP53* ablation resulted in obvious augmentation of the bone tumor burden in hind limbs (Figures 1B,C). Repeated experiments and subsequent survival analysis revealed that intracardiac inoculation with C4-2B/*TP53*-KO cells resulted in a bone metastatic frequency of 87.5%, while that with the C4-2B-vector cells showed a bone metastasis frequency of only 37.5% during a 12-weeks observation period (Figure 1D). Moreover, *TP53*-KO in C4-2B cells significantly increased the number of metastatic foci in the bones (Figure 1E). In addition, *TP53*-KO dramatically accelerated the onset of bone metastasis and greatly reduced the overall survival time in the mice (Figure 1F). These results support that wt-p53 deletion facilitates prostate cancer bone-metastatic capacity *in vivo*.

### Depletion of Wt-p53 Increases Prostate Cancer Cell Chemotaxis to Osteoblasts

**FIGURE 2:**
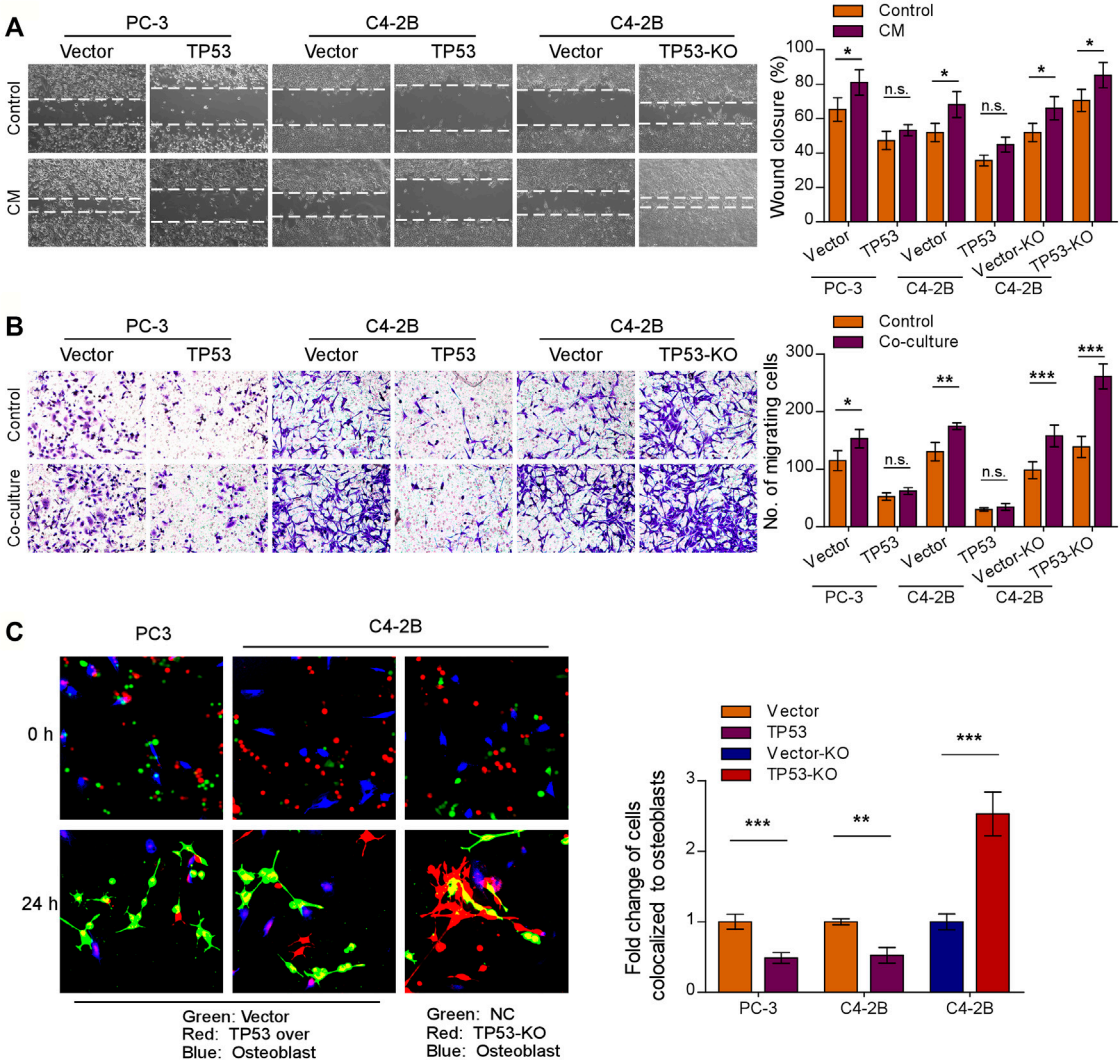
Depletion of wt-p53 increases prostate cancer cells chemotaxis to osteoblasts. **(A)** Representative images (left panel) and quantification (right panel) of wound-healing assays for the indicated cell lines. Wound closure was photographed at 24 h after wounding. Prostate cancer cells were cultured in MC3T3-E1 condition medium (CM). **(B)** Representative micrographs (left panel) and quantification (right panel) of the migration of indicated cells in transwell assays. Co-culture, tumor cells (top chamber) were co-cultured with primary osteoblasts (bottom chamber) in the transwell plate. **(C)** Representative images (left panel) and quantification (right panel) of *in vitro* competitive colocalization assays for the indicated cells by confocal microscopy. Error bars represent the means ± SD of 3 independent experiments. *p < 0.05, **p < 0.01, ***p < 0.001, n. s., no significance. Original magnification, A, ×100; B, ×200; C, ×400.

Initial trafficking and anchoring of tumor cells to the bone marrow endosteal osteoblastic niche is an essential step in the bone metastatic cascade in prostate cancer disease (Haider et al., 2014; Chen F. et al., 2021). To characterize the role of wt-p53 in the chemotaxis of prostate cancer cells to the bone microenvironment, we conducted a series of *in vitro* functional assays. We observed that *TP53*-KO cells had a faster migration rate than the control tumor cells, whereas *TP53* overexpression showed the opposite effect (Figures 2A,B, left upper panel); the pro-migration function of *TP53* depletion on prostate cancer cells was more remarkable when stimulated by osteoblast conditioned medium (Figure 2A). Moreover, when the prostate cancer cell lines C4-2B and PC-3 were placed in the upper chamber and the MC3T3-E1 pre-osteoblasts cells in the lower chamber (as a stimulant in a standard transwell chemotaxis assay), we found that overexpression of *TP53* reduced, while ablation of *TP53* increased the chemotaxis of prostate cancer cells toward the osteoblasts in the lower compartment (Figure 2B). Next, the chemotactic interactions between the cancer cells and bone marrow osteoblasts were further evaluated through *in vitro* competitive colocalization assays, in which metastatic prostate cancer cells were added into and co-cultured with MC3T3-E1 pre-osteoblasts. Significantly, more *TP53*-KO prostate cancer cells were bound and attached to the osteoblasts than control cells (Figure 2C). These results indicate that wt-p53 regulates the chemotaxis of metastatic cancer cells toward bone marrow.

### Chemokine Receptor CXCR4 is a Functional Target of Wt-p53 in Prostate Cancer Bone Metastasis

**FIGURE 3:**
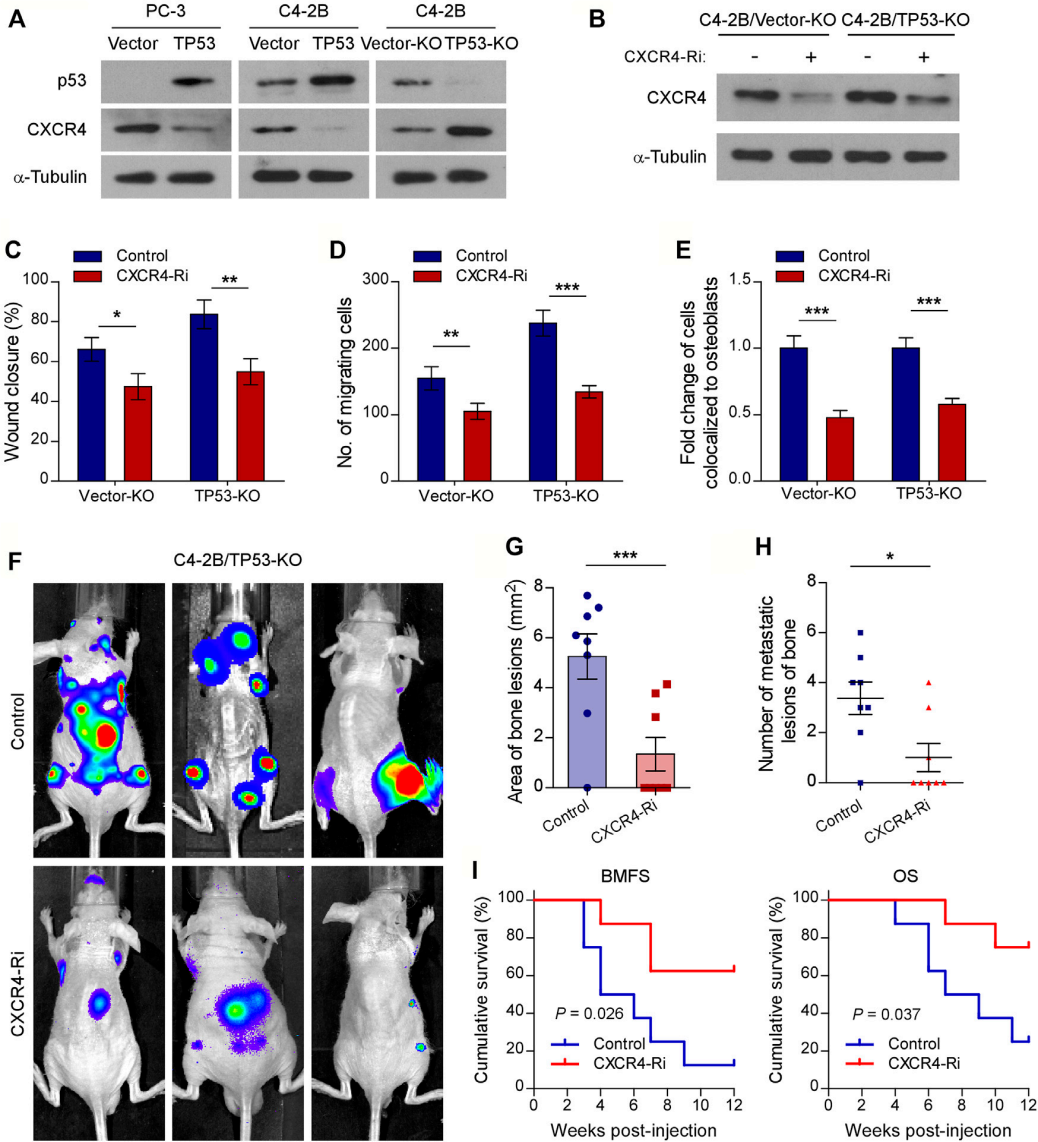
Chemokine receptor CXCR4 is functionally essential target of wt-p53 in prostate cancer bone metastasis. **(A, B)** Western blot analysis of CXCR4 expression in the indicated cells. α-Tubulin served as a loading control. **(C)** Wound-healing assays for the indicated cell lines. Wound closure was photographed at 24 h after wounding. Tumor cells were cultured in MC3T3-E1 condition medium. **(D)** Quantification of the migration of indicated cells in transwell assays. Tumor cells (top chamber) were co-cultured with primary osteoblasts (bottom chamber) in the transwell plate. **(E)** Quantification of *in vitro* competitive colocalization assays for the indicated cells. Error bars represent the means ± SD. **(F)** Representative BLI images of bone lesions from mice intracardially inoculated with prostate cancer cells in each experimental group (*n* = 8/group). **(G)** Histomorphometric quantification of tumor area in hind limbs from each experimental group. **(H)** Numbers of metastatic lesions in hind limb bones from each mouse in two groups. Error bars represent the means ± SEM. **(I)** Kaplan-Meier curves of bone metastasis-free survival (BMFS) and overall survival (OS) in mice from each experimental group (log-rank test). *P* values were based on *t*-test unless otherwise indicated. *p < 0.05, **p < 0.01, ***p < 0.001.

Previous studies have shown that the CXCL12/CXCR4 chemokine axis plays a critical role in localizing tumor cells to the bone marrow during the early stages of bone metastasis in prostate cancer (Sun et al., 2005; Shiozawa et al., 2011). To investigate the molecular mechanisms of wt-p53 function in bone metastasis, we examined the involvement of wt-p53 in the regulation of CXCR4/CXCL12 chemokine axis. Western blot analysis showed that the protein levels of CXCR4 dramatically upregulated in *TP53*-KO cells but reduced in *TP53*-overexpressing cells, compared with control cells (Figure 3A). We next investigated whether the pro-bone metastatic effects of *TP53* deletion are mediated by the CXCR4/CXCL12 signaling axis activation. Silencing of CXCR4 suppressed the migration rate, chemotaxis, and attachment of C4-2B cells toward the osteoblasts *in vitro* (Figures 3B–E). Importantly, *TP53*-KO-induced tumor aggression was significantly impaired by CXCR4 ablation *in vitro* (Figures 3C–E). Moreover, silencing of CXCR4 in *TP53*-deleted C4-2B cells decreased the burden of bone metastasis in the tumor-bearing mice (Figures 3F,G). Repeated experiments and subsequent survival analysis revealed that CXCR4 silencing significantly decreased the occurrence of bone metastasis, delayed the onset of bone metastasis, and prolonged overall survival of mice inoculated intracardially with *TP53* deletion-C4-2B cells (Figures 3H,I). These findings suggest that CXCR4/CXCL12 signaling plays an important role in promoting the metastasis of prostate cancer cells to bone, whereas *TP53* controls the chemotaxis of cancer cells to the bone marrow endosteal osteoblastic niche by modulating CXCR4 expression.

### Wt-p53 Transcriptionally Upregulates AIP4, a Negatively Regulator of CXCL12/CXCR4 Signaling

**FIGURE 4:**
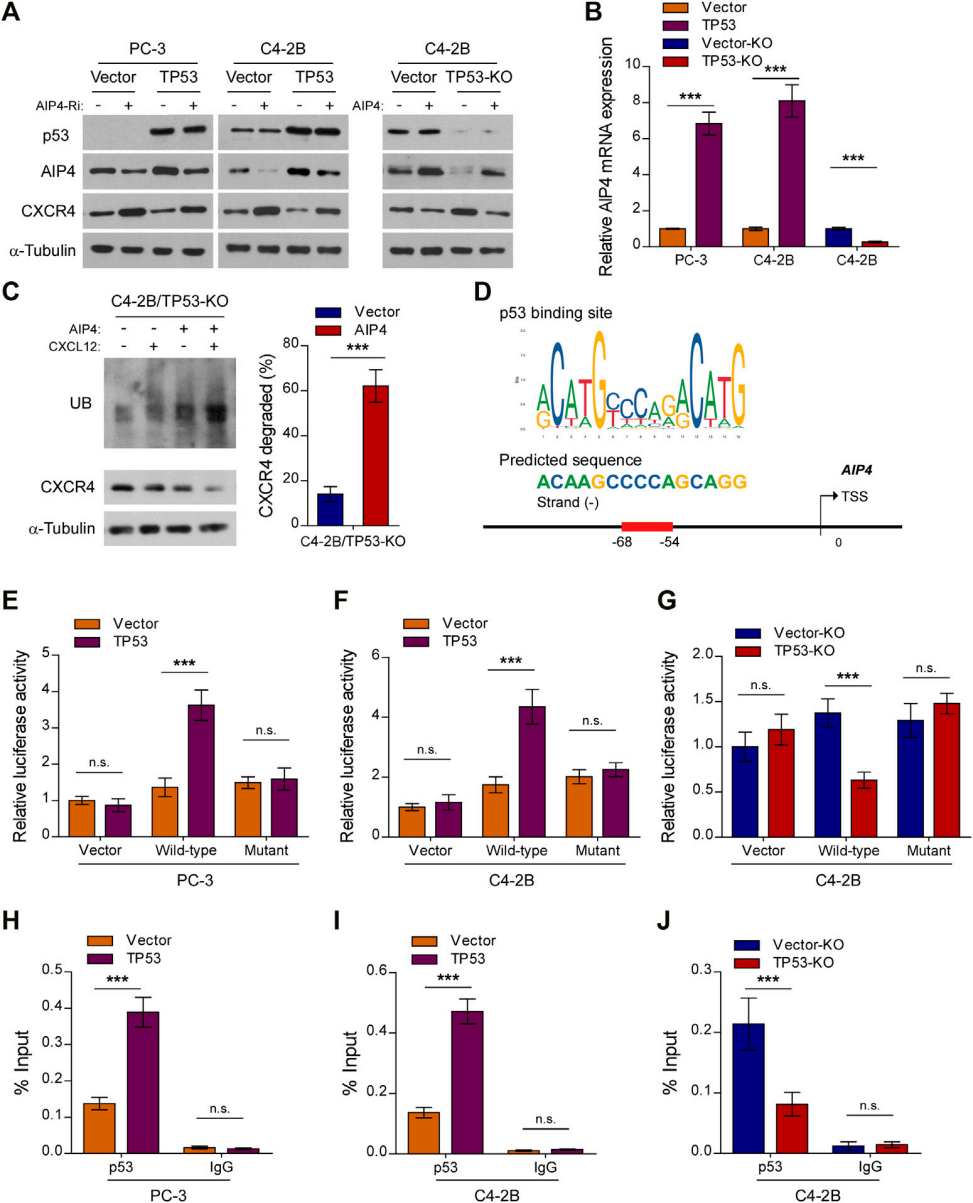
Wt-p53 transcriptionally upregulates AIP4, a negatively regulator of CXCL12/CXCR4 signaling. **(A)** Western blot analysis of CXCR4 and AIP4 expression in the indicated cells. α-Tubulin served as a loading control. **(B)** Real-time PCR analysis of *AIP4* mRNA expression in the indicated cells. Transcript levels were normalized to GAPDH. **(C)** Ubiquitination of CXCR4 was detected by Western blot. C4-2B/*TP53*-KO cells transfected with HA-tagged *CXCR4* (1 µg) and 3×FLAG-tagged ubiquitin (1 µg) plus either empty vector or *AIP4* plasmid, were treated with or without CXCL12. Cell lysates were incubated with an anti-HA antibody and the immunoprecipitates were analyzed by SDS-PAGE followed by western blot using an anti-FLAG antibody. Total cell lysates were also subjected to immunoblotting to detect the expression of CXCR4. α-Tubulin was used as loading controls. Right panel: quantification of relative CXCR4 degradation. **(D)** Schematic illustration of the predicted binding site for p53 in the indicated *AIP4* promoter regions. **(E–G)** Quantification of luciferase activity of the *AIP4* promoter reporter (with wild-type or mutant putative p53-binding sequences) in the indicated cells. **(H–J)** ChIP analysis of enrichment of p53 on the *AIP4* gene promoter. IgG was used as a negative control. Error bar represents the mean ± SD of 3 independent experiments. *p < 0.05, **p < 0.01, ***p < 0.001, n. s., no significance.

Upon CXCL12 ligand stimulation, CXCR4 is phosphorylated, ubiquitinated and subsequently sorted into the lysosomal degradative pathway (Caballero et al., 2019). The E3 ubiquitin ligase AIP4 is responsible for the ubiquitination-mediated CXCR4 protein degradation and modification process (Marchese et al., 2003). Interestingly, qRT-PCR and western blot analysis revealed that the expression and level of AIP4, respectively, was dramatically reduced in *TP53*-ablation cells, but increased in *TP53*-overexpressing cells, as compared with control cells (Figures 4A,B). Moreover, *TP53* deletion-induced overexpression of CXCR4 was abrogated by co-expression of *AIP4*, and *AIP4* siRNA could rescue CXCR4 inhibition by *TP53* (Figure 4A). In addition, we found that co-expression of *AIP4* resulted in a significant accumulation of ubiquitinated CXCR4 and subsequently suppressed CXCR4 protein levels, indicating that AIP4 inhibits the ligand-induced degradation of CXCR4 in *TP53*-deleted prostate cancer cells (Figure 4C). Analyses using the JASPAR and Genome Browser Gateway website programs predicted wt-p53 specific binding sites in the region (−68, −54) of the *AIP4* promoter (Figure 4D), suggesting that p53 might transcriptionally regulate *AIP4* by binding to its promoter regions. Luciferase reporter assays confirmed that overexpression of *TP53* enhanced the luciferase activity driven by the *AIP4* promoters, whereas deletion of *TP53* attenuated this activity (Figures 4E–G). Nevertheless, altered expression of wt-p53 did not affect luciferase activities of *AIP4* promoters that contained mutated p53-specific binding sites (Figures 4E–G). Furthermore, ChIP assays revealed that wt-p53 was capable of binding to the *AIP4* gene promoter regions (Figures 4H–J). These results suggest that wt-p53 could facilitate the upregulation of AIP4 in prostate cancer by directly binding to the promoter of the *AIP4* gene to promote its transcription.

### CXCR4 Inhibitor or *AIP4* Overexpression Reverses the Pro-bone Metastasis Effects of TP53 Deletion

**FIGURE 5:**
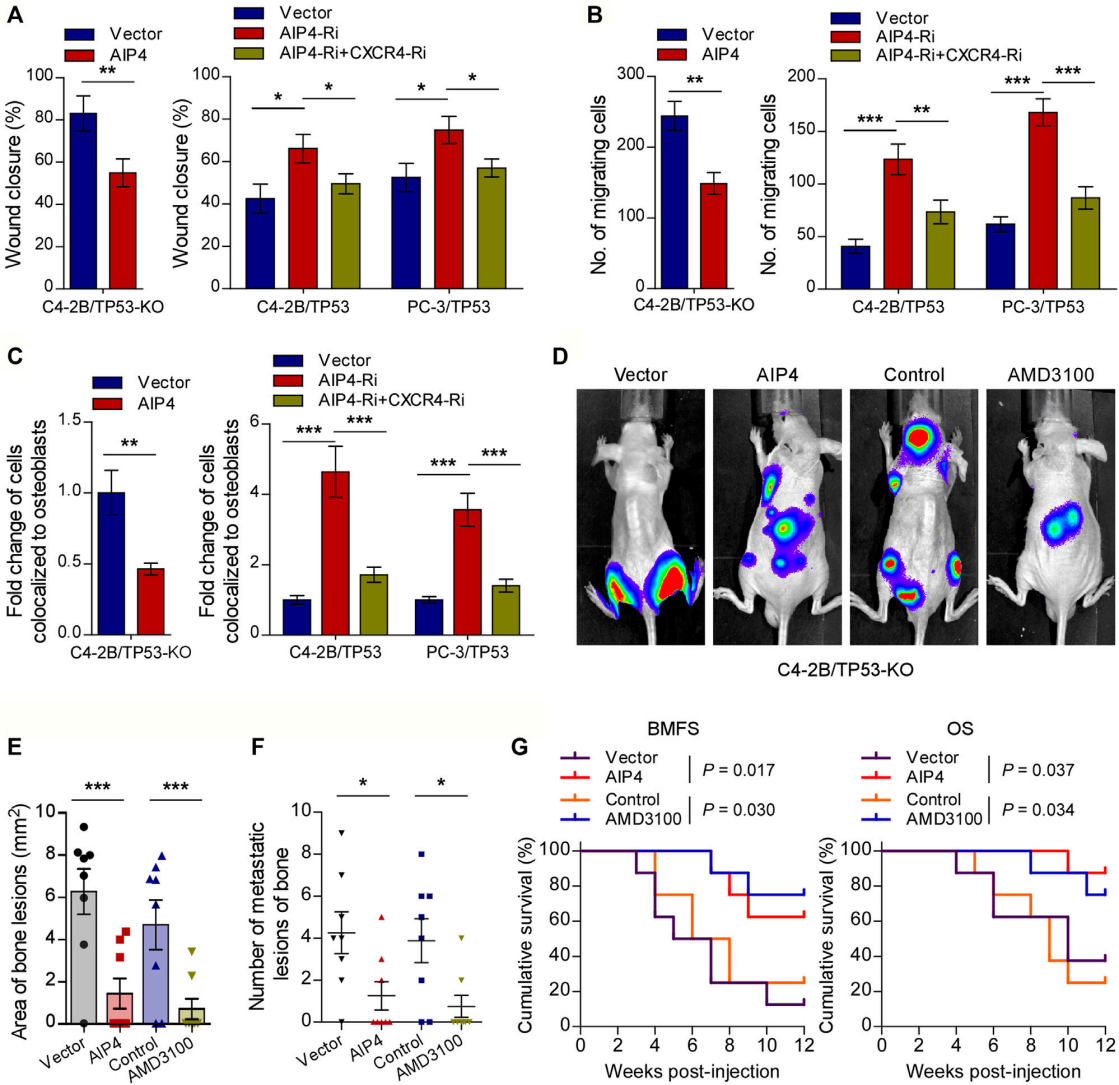
CXCR4 inhibitor and *AIP4* plasmid transduction reverse the pro-bone metastasis effects of *TP53* deletion. **(A)** Wound-healing assays for the indicated cell lines. Wound closure was photographed at 24 h after wounding. Tumor cells were cultured in MC3T3-E1 condition medium. **(B)** Quantification of the migration of indicated cells in transwell assays. Tumor cells (top chamber) were co-cultured with primary osteoblasts (bottom chamber) in the transwell plate. **(C)** Quantification of *in vitro* competitive colocalization assays for the indicated cells. Error bars represent the means ± SD. **(D)** Representative BLI images of bone lesions from mice inoculated intracardially with indicated tumor cells in each experimental group (*n* = 8/group). Mice were administered with intraperitoneal injection of AMD3100 (Plerixafor, AbMole, 5 mg/kg, every 2 days). **(E)** Histomorphometric quantification of tumor area in hind limbs from each experimental group. **(F)** Numbers of metastatic lesions in hind limb bones from each mouse in two groups. Error bars represent the means ± SEM. **(G)** Kaplan-Meier curves of bone metastasis-free survival (BMFS) and overall survival (OS) in mice from each experimental group (log-rank test). *p < 0.05. *P* values were based on *t*-test unless otherwise indicated.

We next evaluated whether the pro-bone metastasis functions of *TP53* deletion in prostate cancer were mediated by *AIP4* downregulation and CXCL12/CXCR4 axis activation. As expected, the results of *in vitro* experiments indicated that *AIP4* co-expression significantly suppressed the *TP53*-deletion prostate cancer cells migration rate, chemotaxis, and attachment toward the osteoblasts (Figures 5A–C). Inversely, *AIP4* silencing notably reversed the anti-migration and -chemotaxis effects of *TP53* on tumor cells, while co-expression with *CXCR4* siRNA abrogated these aggressive tumor behaviors in *TP53* overexpressing tumor cells (Figures 5A–C). Moreover, *in vivo* studies showed that transduction of *AIP4* plasmid DNA into tumor cells or treatment with AMD3100, a selective CXCR4 receptor inhibitor, significantly suppressed the number of bone metastatic lesions and tumor burden in hind limbs, retarded the onset of bone metastasis, and markedly prolonged the survival time in the tumor-bearing mice (Figures 5D–G).

### The Levels of Wt-p53, AIP4, and CXCR4 Were Clinically Relevant in Human Prostate Cancer

**FIGURE 6:**
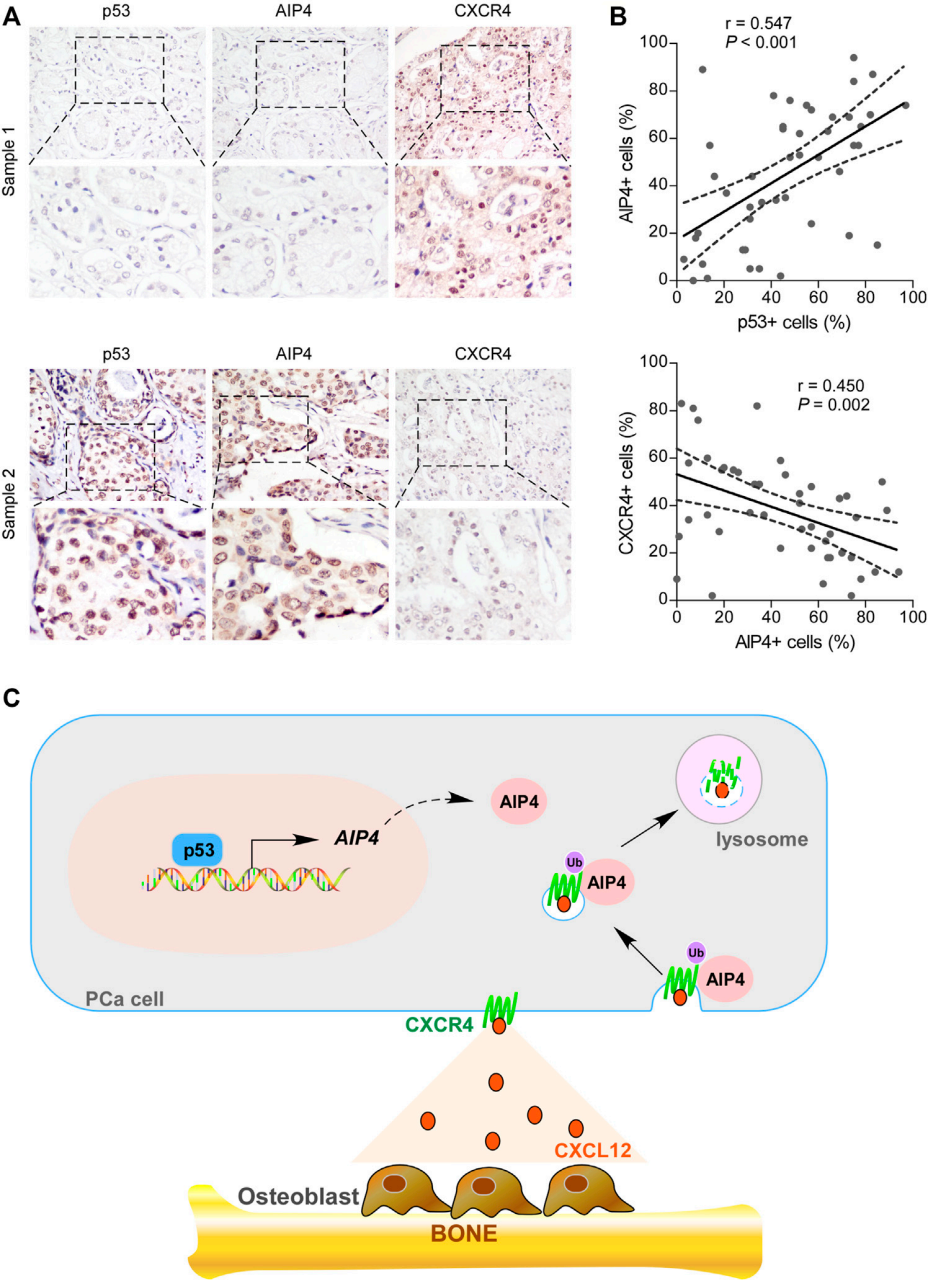
The expression of wt-p53, AIP4, and CXCR4 were clinically relevant in human prostate cancer. **(A)** Representative images of wt-p53, AIP4, and CXCR4 staining in prostate cancer patient specimens. Two representative cases are shown. Original magnification, up panel: ×200; down panel: inset. **(B)** Pearson’s correlation analysis of protein expression between genes based on IHC data of prostate cancer specimens. **(C)** Model: p53 transcriptionally activates the downstream ubiquitin ligase AIP4 to promote degradation of the chemokine receptor CXCR4, and thus blocks the trafficking of CXCR4-positive prostate cancer cells to the bone marrow.

We further examined whether the wt-p53/AIP4/CXCR4 axis identified in human prostate cancer cells was clinically relevant by conducting IHC analysis. As shown in Figures 6A,B, the protein levels of wt-p53 were positively correlated with the levels of AIP4 (*p* < 0.001; *n* = 46), while inversely correlated with the CXCR4 levels (*p* = 0.002; *n* = 46). Collectively, these results indicate that inhibition of wt-p53 in human prostate cancer reduces AIP4 levels to activate CXCR4/CXCL12 axis, ultimately leading to tumor cell bone metastasis (Figure 6C).

## Discussion

*TP53* is frequently mutated in human cancers (Hainaut et al., 1998). Loss of wt-p53 and gain-of-function *TP53* mutation confer on tumor cells a greater propensity for metastasis to distant sites (Tang et al., 2020). Our group has been committed to elucidating the role of wt-p53 in prostate cancer bone metastases and how it mediates the molecular mechanism. We previously found that wt-p53 suppresses EMT, self-renewal capability, and colony formation of prostate cancer PC-3 cells, at least partially, by modulating the miR-145 expression (Ren et al., 2013). Moreover, Frizzled-8 receptor (FZD8) activated Wnt/β-catenin signaling promoted prostate cancer cell migration, invasion, and stem cell-like phenotypes *in vitro*, and enhanced bone metastasis *in vivo* (Li et al., 2017b). Likewise, FZD8 was a direct target of wt-p53. These results are consistent with previous reports indicating that wt-p53 regulates multiple steps of the metastatic cascade through modulating a variety of metastasis-related genes (Tang et al., 2020). Yet, direct evidence for the role of p53 in skeletal metastasis and the molecular mechanism by which p53 regulates the bone metastatic propensity of tumor cells remains obscure. In this study, we revealed that depletion of wt-p53 in prostate cancer cells favored early steps of bone metastatic homing and colonization, conferring chemotaxis and attachment of prostate cancer cells to osteoblasts, through increasing the activity of the CXCR4/CXCL12 chemokine axis. Therefore, targeting the wt-p53/CXCR4/CXCL12 signaling might be an appealing therapeutic concept for the treatment of metastatic bone disease, which requires further clinical investigation.

The chemokine receptor CXCR4 is aberrantly expressed in various solid human cancers (Teixidó et al., 2018). Overexpression of CXCR4 has been implicated in aggressive and metastatic tumor phenotypes with poor clinical prognosis (Katsura et al., 2018). The expression of CXCR4 is influenced by numerous factors, such as hypoxic conditions, stress, as well as transcriptional and post-translational modifications (Kuo et al., 2018; Caballero et al., 2019; Chen YY. et al., 2021). In the present study, we found that the protein levels of CXCR4 were dramatically increased, while the expression of AIP4 was reduced, in TP53-ablation cells. Co-expression of *AIP4* resulted in a significant accumulation of ubiquitinated CXCR4 and subsequently suppressed CXCR4 protein levels in *TP53* deletion prostate cancer cells. These results supported that deletion of wt-p53 promoted degradation of CXCR4, at least partially, by modulating the expression of *AIP4*. Interestingly, Mehta and colleagues have revealed that in breast cancer cells, wt-p53 could indirectly and negatively regulate the expression of *CXCR4* through inducing the binding of the transcription factors ATF-1 and c-Jun to a cyclic AMP/AP-1 response like element in the *CXCR4* promoter (Mehta et al., 2007). Thus, these results expand our understanding of the dysregulation of CXCR4 expression in prostate cancer progression, suggesting that negative regulation of CXCR4 signaling by wt-p53 might be mediated through both transcriptional and post-translational mechanisms.

Previous studies have reported that many factors and pathways might be involved in CXCR4 degradation. The E3 ubiquitin ligase AIP4 is responsible for agonist-dependent ubiquitination of CXCR4 at the plasma membrane, and mediates consequent endosomal sorting and degradation of the receptor (Marchese et al., 2003). The scaffold proteins arrestin-2 could interact and colocalize with AIP4 on early endosomes, while depletion of arrestin-2 impedes agonist-dependent degradation of CXCR4 by preventing CXCR4 trafficking from early endosomes to lysosomes (Bhandari et al., 2007). Moreover, STAM-1 interacts directly with arrestin-2 to negatively modulate CXCR4 endosomal sorting by regulating the ubiquitination status of HRS (Malik and Marchese, 2010). In addition, CXCR4 lysosomal trafficking and degradation are also under heterologous regulation by PKC and GRK6, *via* activation of other GPCRs, such as CXCR5 (Caballero et al., 2019). However, we regret that we did not check the role of PKC, GRK6, arrestin-2, STAM-1, and HRS in the p53/AIP4/CXCR4 axis. The relationships between wt-p53 and these factors in the regulation of CXCR4 degradation needs future investigation.

AIP4 is a member of the Nedd4 family of E3 ligases that promote the ubiquitination of a variety of substrates (Lorenz, 2018). Numerous studies have demonstrated that AIP4 can acts as a double-edged sword in cancer progression (Yin et al., 2020). Lim and colleagues found that silencing of AIP4 blocks ubiquitin-dependent proteasomal degradation of the YAP/TAZ transcriptional coactivator WBP2 as well as promotes anchorage-independent colony and *in vivo* tumor growth in breast cancer (Lim et al., 2016). Moreover, activation of AIP4 interacts with and induces Gli1 degradation, while mutation of AIP4-dependent degron in Gli1 enhances medulloblastoma growth, migration, invasion, and *in vitro* transforming activity, suggesting AIP4 functions as a tumor suppressor (Di Marcotullio et al., 2011). Nevertheless, Ho and colleagues discovered that AIP4 promotes degradation of LATS1 to enhance nuclear translocation of YAP and thus increases breast cancer cell proliferation while reducing apoptosis (Ho et al., 2011). Wild-type AIP4 induces tumor poly-ubiquitination and degradation of suppressor protein RASSF5 to suppress RASSF5A-mediated G1 arrest and apoptosis in HEK293T cells (Suryaraja et al., 2013). These data reveal that AIP4 exhibits both tumor suppressive and oncogenic properties under different tissue types and cellular conditions, largely depending on the downstream substrates. Our data indicate that p53-induced AIP4 promoted CXCR4 ubiquitination to reduce its protein level and consequently blocked the metastasis of prostate cancer cells to the bone, suggesting AIP4 is a tumor suppressor in prostate cancer.

In summary, this work identified the wt-p53/AIP4/CXCR4 axis as a novel pathway facilitating the colonization of prostate tumor cells in the bone. Furthermore, we demonstrated that therapeutic intervention targeting this pathway suppresses tumor cells migration rate, chemotaxis and attachment toward the osteoblasts *in vitro*, and prevents disseminated tumor cells metastasis to the bone *in vivo*. Therefore, our results provide a strong rationale for the inhibition of this pathway to prevent metastatic tumor cell colonization in the bones of patients with prostate cancer.

## Data Availability

The raw data supporting the conclusions of this article will be made available by the authors, without undue reservation.

## References

[B1] Bhandari D. Trejo J. Benovic J. L. Marchese A. (2007). Arrestin-2 Interacts with the Ubiquitin-Protein Isopeptide Ligase Atrophin-Interacting Protein 4 and Mediates Endosomal Sorting of the Chemokine Receptor CXCR4. J. Biol. Chem. 282 (51), 36971–36979. 10.1074/jbc.M705085200 17947233

[B2] Broxmeyer H. E. Orschell C. M. Clapp D. W. Hangoc G. Cooper S. Plett P. A. (2005). Rapid Mobilization of Murine and Human Hematopoietic Stem and Progenitor Cells with AMD3100, a CXCR4 Antagonist. J. Exp. Med. 201 (8), 1307–1318. 10.1084/jem.20041385 15837815 PMC2213145

[B3] Caballero A. Mahn S. A. Ali M. S. Rogers M. R. Marchese A. (2019). Heterologous Regulation of CXCR4 Lysosomal Trafficking. J. Biol. Chem. 294 (20), 8023–8036. 10.1074/jbc.RA118.005991 30936203 PMC6527173

[B4] Chen F. Han Y. Kang Y. (2021). Bone Marrow Niches in the Regulation of Bone Metastasis. Br. J. Cancer. 124 (12), 1912–1920. 10.1038/s41416-021-01329-6 33758331 PMC8184962

[B5] Chen Y. Y. Liu Y. F. Liu Y. D. Deng X. H. Zhou J. (2021). IRF7 Suppresses Hematopoietic Regeneration under Stress via CXCR4. Stem Cells. 39 (2), 183–195. 10.1002/stem.3308 33252829

[B6] Clarke N. W. Hart C. A. Brown M. D. (2009). Molecular Mechanisms of Metastasis in Prostate Cancer. Asian J. Androl. 11 (1), 57–67. 10.1038/aja.2008.29 19050684 PMC3735202

[B7] Dar A. Schajnovitz A. Lapid K. Kalinkovich A. Itkin T. Ludin A. (2011). Rapid Mobilization of Hematopoietic Progenitors by AMD3100 and Catecholamines Is Mediated by CXCR4-Dependent SDF-1 Release from Bone Marrow Stromal Cells. Leukemia. 25 (8), 1286–1296. 10.1038/leu.2011.62 21494253 PMC4175714

[B8] Di Marcotullio L. Greco A. Mazzà D. Canettieri G. Pietrosanti L. Infante P. (2011). Numb Activates the E3 Ligase Itch to Control Gli1 Function Through a Novel Degradation Signal. Oncogene. 30 (1), 65–76. 10.1038/onc.2010.394 20818436

[B9] Festuccia C. Mancini A. Gravina G. L. Colapietro A. Vetuschi A. Pompili S. (2019). Dual CXCR4 and E-Selectin Inhibitor, GMI-1359, Shows Anti-bone Metastatic Effects and Synergizes with Docetaxel in Prostate Cancer Cell Intraosseous Growth. Cells. 9 (1), 32. 10.3390/cells9010032 PMC7017374 31877673

[B10] Gandaglia G. Abdollah F. Schiffmann J. Trudeau V. Shariat S. F. Kim S. P. (2014). Distribution of Metastatic Sites in Patients with Prostate Cancer: A Population-Based Analysis. Prostate. 74 (2), 210–216. 10.1002/pros.22742 24132735

[B11] Grishagin I. V. (2015). Automatic Cell Counting with ImageJ. Anal. Biochem. 473, 63–65. 10.1016/j.ab.2014.12.007 25542972

[B12] Haider M. T. Holen I. Dear T. N. Hunter K. Brown H. K. (2014). Modifying the Osteoblastic Niche with Zoledronic Acid In Vivo-Potential Implications for Breast Cancer Bone Metastasis. Bone. 66 (100), 240–250. 10.1016/j.bone.2014.06.023 24971713 PMC4127787

[B13] Hainaut P. Hernandez T. Robinson A. Rodriguez-Tome P. Flores T. Hollstein M. (1998). IARC Database of P53 Gene Mutations in Human Tumors and Cell Lines: Updated Compilation, Revised Formats and New Visualisation Tools. Nucleic Acids Res. 26 (1), 205–213. 10.1093/nar/26.1.205 9399837 PMC147235

[B14] Ho K. C. Zhou Z. She Y. M. Chun A. Cyr T. D. Yang X. (2011). Itch E3 Ubiquitin Ligase Regulates Large Tumor Suppressor 1 Stability [corrected]. Proc. Natl. Acad. Sci. U. S. A. 108 (12), 4870–4875. 10.1073/pnas.1101273108 21383157 PMC3064387

[B15] Ito K. (2014). Prostate Cancer in Asian Men. Nat. Rev. Urol. 11 (4), 197–212. 10.1038/nrurol.2014.42 24595118

[B16] Kastenhuber E. R. Lowe S. W. (2017). Putting P53 in Context. Cell. 170 (6), 1062–1078. 10.1016/j.cell.2017.08.028 28886379 PMC5743327

[B17] Katsura M. Shoji F. Okamoto T. Shimamatsu S. Hirai F. Toyokawa G. (2018). Correlation between CXCR4/CXCR7/CXCL12 Chemokine axis Expression and Prognosis in Lymph-Node-Positive Lung Cancer Patients. Cancer Sci. 109 (1), 154–165. 10.1111/cas.13422 29032612 PMC5765305

[B18] Kucia M. Reca R. Miekus K. Wanzeck J. Wojakowski W. Janowska-Wieczorek A. (2005). Trafficking of normal Stem Cells and Metastasis of Cancer Stem Cells Involve Similar Mechanisms: Pivotal Role of the SDF-1-CXCR4 axis. Stem Cells. 23 (7), 879–894. 10.1634/stemcells.2004-0342 15888687

[B19] Kuo Y. C. Au H. K. Hsu J. L. Wang H. F. Lee C. J. Peng S. W. (2018). IGF-1R Promotes Symmetric Self-Renewal and Migration of Alkaline Phosphatase+ Germ Stem Cells Through HIF-2α-Oct4/Cxcr4 Loop Under Hypoxia. Stem Cell Rep. 10 (2), 524–537. 10.1016/j.stemcr.2017.12.003 PMC5830933 29307582

[B20] Li Q. Wang M. Hu Y. Zhao E. Li J. Ren L. (2021). MYBL2 Disrupts the Hippo-YAP Pathway and Confers Castration Resistance and Metastatic Potential in Prostate Cancer. Theranostics. 11 (12), 5794–5812. 10.7150/thno.56604 33897882 PMC8058714

[B21] Li Q. Ye L. Guo W. Wang M. Huang S. Peng X. (2017a). PHF21B Overexpression Promotes Cancer Stem Cell-Like Traits in Prostate Cancer Cells by Activating the Wnt/β-Catenin Signaling Pathway. J. Exp. Clin. Cancer Res. 36 (1), 85. 10.1186/s13046-017-0560-y 28645312 PMC5481925

[B22] Li Q. Ye L. Zhang X. Wang M. Lin C. Huang S. (2017b). FZD8, a Target of P53, Promotes Bone Metastasis in Prostate Cancer by Activating Canonical Wnt/β-Catenin Signaling. Cancer Lett. 402, 166–176. 10.1016/j.canlet.2017.05.029 28602974

[B23] Lim S. K. Lu S. Y. Kang S. A. Tan H. J. Li Z. Adrian Wee Z. N. (2016). Wnt Signaling Promotes Breast Cancer by Blocking ITCH-Mediated Degradation of YAP/TAZ Transcriptional Coactivator WBP2. Cancer Res. 76 (21), 6278–6289. 10.1158/0008-5472.can-15-3537 27578003

[B24] Lorenz S. (2018). Structural Mechanisms of HECT-type Ubiquitin Ligases. Biol. Chem. 399 (2), 127–145. 10.1515/hsz-2017-0184 29016349

[B25] Malik R. Marchese A. (2010). Arrestin-2 Interacts with the Endosomal Sorting Complex Required for Transport Machinery to Modulate Endosomal Sorting of CXCR4. Mol. Biol. Cell. 21 (14), 2529–2541. 10.1091/mbc.e10-02-0169 20505072 PMC2903679

[B26] Marchese A. Raiborg C. Santini F. Keen J. H. Stenmark H. Benovic J. L. (2003). The E3 Ubiquitin Ligase AIP4 Mediates Ubiquitination and Sorting of the G Protein-Coupled Receptor CXCR4. Dev. Cell. 5 (5), 709–722. 10.1016/s1534-5807(03)00321-6 14602072

[B27] Mehta S. A. Christopherson K. W. Bhat-Nakshatri P. Goulet R. J. Jr. Broxmeyer H. E. Kopelovich L. (2007). Negative Regulation of Chemokine Receptor CXCR4 by Tumor Suppressor P53 in Breast Cancer Cells: Implications of P53 Mutation or Isoform Expression on Breast Cancer Cell Invasion. Oncogene. 26 (23), 3329–3337. 10.1038/sj.onc.1210120 17130833

[B28] Müller A. Homey B. Soto H. Ge N. Catron D. Buchanan M. E. (2001). Involvement of Chemokine Receptors in Breast Cancer Metastasis. Nature. 410 (6824), 50–56. 10.1038/35065016 11242036

[B29] Network C. G. A. R. (2015). The Molecular Taxonomy of Primary Prostate Cancer. Cell. 163 (4), 1011–1025. 10.1016/j.cell.2015.10.025 26544944 PMC4695400

[B30] Nørgaard M. Jensen A. Ø. Jacobsen J. B. Cetin K. Fryzek J. P. Sørensen H. T. (2010). Skeletal Related Events, Bone Metastasis and Survival of Prostate Cancer: a Population Based Cohort Study in Denmark (1999 to 2007). J. Urol. 184 (1), 162–167. 10.1016/j.juro.2010.03.034 20483155

[B31] Park J. W. Lee J. K. Sheu K. M. Wang L. Balanis N. G. Nguyen K. (2018). Reprogramming Normal Human Epithelial Tissues to a Common, Lethal Neuroendocrine Cancer Lineage. Science. 362 (6410), 91–95. 10.1126/science.aat5749 30287662 PMC6414229

[B32] Puca R. Nardinocchi L. Gal H. Rechavi G. Amariglio N. Domany E. (2008). Reversible Dysfunction of Wild-type P53 Following Homeodomain-Interacting Protein Kinase-2 Knockdown. Cancer Res. 68 (10), 3707–3714. 10.1158/0008-5472.CAN-07-6776 18483253

[B33] Ren D. Wang M. Guo W. Zhao X. Tu X. Huang S. (2013). Wild-type P53 Suppresses the Epithelial-Mesenchymal Transition and Stemness in PC-3 Prostate Cancer Cells by Modulating miR-145. Int. J. Oncol. 42 (4), 1473–1481. 10.3892/ijo.2013.1825 23404342

[B34] Robinson D. Van Allen E. M. Wu Y. M. Schultz N. Lonigro R. J. Mosquera J. M. (2015). Integrative Clinical Genomics of Advanced Prostate Cancer. Cell. 161 (5), 1215–1228. 10.1016/j.cell.2015.05.001 26000489 PMC4484602

[B35] Schlechte H. Lenk S. V. Löning T. Schnorr D. Rudolph B. D. Ditscherlein G. (1998). p53 Tumour Suppressor Gene Mutations in Benign Prostatic Hyperplasia and Prostate Cancer. Eur. Urol. 34 (5), 433–440. 10.1159/000019778 9803007

[B36] Shiozawa Y. Pedersen E. A. Havens A. M. Jung Y. Mishra A. Joseph J. (2011). Human Prostate Cancer Metastases Target the Hematopoietic Stem Cell Niche to Establish Footholds in Mouse Bone Marrow. J. Clin. Invest. 121 (4), 1298–1312. 10.1172/jci43414 21436587 PMC3069764

[B37] Siegel R. L. Miller K. D. Jemal A. (2018). Cancer Statistics, 2018. CA Cancer J. Clin. 68 (1), 7–30. 10.3322/caac.21442 29313949

[B38] Sun Y. X. Schneider A. Jung Y. Wang J. Dai J. Wang J. (2005). Skeletal Localization and Neutralization of the SDF-1(CXCL12)/CXCR4 axis Blocks Prostate Cancer Metastasis and Growth in Osseous Sites *In Vivo* . J. Bone Miner. Res. 20 (2), 318–329. 10.1359/jbmr.041109 15647826

[B39] Sung H. Ferlay J. Siegel R. L. Laversanne M. Soerjomataram I. Jemal A. (2021). Global Cancer Statistics 2020: GLOBOCAN Estimates of Incidence and Mortality Worldwide for 36 Cancers in 185 Countries. CA Cancer J. Clin. 71 (3), 209–249. 10.3322/caac.21660 33538338

[B40] Suryaraja R. Anitha M. Anbarasu K. Kumari G. Mahalingam S. (2013). The E3 Ubiquitin Ligase Itch Regulates Tumor Suppressor Protein RASSF5/NORE1 Stability in an Acetylation-Dependent Manner. Cell Death Dis. 4 (3), e565. 10.1038/cddis.2013.91 23538446 PMC3615736

[B41] Tang Q. Su Z. Gu W. Rustgi A. K. (2020). Mutant P53 on the Path to Metastasis. Trends Cancer. 6 (1), 62–73. 10.1016/j.trecan.2019.11.004 31952783 PMC7485681

[B42] Teixidó J. Martínez-Moreno M. Díaz-Martínez M. Sevilla-Movilla S. (2018). The Good and Bad Faces of the CXCR4 Chemokine Receptor. Int. J. Biochem. Cell Biol. 95, 121–131. 10.1016/j.biocel.2017.12.018 29288743

[B43] Väyrynen J. P. Vornanen J. O. Sajanti S. Böhm J. P. Tuomisto A. Mäkinen M. J. (2012). An Improved Image Analysis Method for Cell Counting Lends Credibility to the Prognostic Significance of T Cells in Colorectal Cancer. Virchows Arch. 460 (5), 455–465. 10.1007/s00428-012-1232-0 22527018

[B44] Ye L. Lin C. Wang X. Li Q. Li Y. Wang M. (2019). Epigenetic Silencing of SALL2 Confers Tamoxifen Resistance in Breast Cancer. EMBO Mol. Med. 11 (12), e10638. 10.15252/emmm.201910638 31657150 PMC6895605

[B45] Yin Q. Wyatt C. J. Han T. Smalley K. S. M. Wan L. (2020). ITCH as a Potential Therapeutic Target in Human Cancers. Semin. Cancer Biol. 67 (Pt 2), 117–130. 10.1016/j.semcancer.2020.03.003 32165318 PMC7724637

[B46] Yoneda T. Hiraga T. (2005). Crosstalk Between Cancer Cells and Bone Microenvironment in Bone Metastasis. Biochem. Biophys. Res. Commun. 328 (3), 679–687. 10.1016/j.bbrc.2004.11.070 15694401

