# Supplementary material for: Deletion of Wild-type p53 Facilitates Bone Metastatic Function by Blocking the AIP4 Mediated Ligand-Induced Degradation of CXCR4
Source: Front Pharmacol. 2022 Feb 1;12:792293. doi: 10.3389/fphar.2021.792293 (PMC8844016; doi:10.3389/fphar.2021.792293)
Supplement: Supplementary file 1 [file Table1.pdf]

Table S1. Clinicopathological characteristics of PCa patients

Characteristics		Total (46)
Age (years)	≤ 73	22
	> 73	24
Total PSA (ng/ml)	≤ 10	17
	> 10	29
Gleason score	≤ 7	19
	> 7	27
T stage	T2	30
	T3-4	16
N stage	N0	32
	N1	14
Relapse/Metastasis status	Negative	35
	Positive	11
