# Supplementary material for: Deletion of Wild-type p53 Facilitates Bone Metastatic Function by Blocking the AIP4 Mediated Ligand-Induced Degradation of CXCR4
Source: Front Pharmacol. 2022 Feb 1;12:792293. doi: 10.3389/fphar.2021.792293 (PMC8844016; doi:10.3389/fphar.2021.792293)
Supplement: Supplementary file 2 [file Table2.pdf]

Table S2. The sequences of the *AIP4* promoter and the mutants driving the luciferase promoter.

Name	Location	Sequence
Widetype	hg38_dna<br>range=chr20:<br>34362774-34<br>363773	GGCCGCCGCCCCTCACAGGGCCACGTCGCCCGCG<br>CCGGCCCCGCCGCCGGGCCCCGCGCGTAGGCCGA<br>ACCGCTGGAGAGTGGCCCCAGGCGGGGCTGAGG<br>GGCGAGGGGCCGGACGGTACCCACGCCCGTCGT<br>GGCCCAGCCCTTTCACAACTTGGAGCCCGCGAGG<br>CGAAGGCCTCCGGAAGCCTGTGCCAGACACTGAT<br>CCCTCCGAACGTCCAGACAGCCCACCCCCACCGC<br>CCGGCCCGGGCCAGTAGCCCGAGCTCCCCGCGCC<br>CCCGACAGACACCGGCTCTGGAAGAAGGAGCAG<br>CCGCGGCTCTGGGGTCCCGAACCGCGCGTCCCAC<br>TCCTCTACCCTTGTTCTCCCCGAACATTCTCATCC<br>GGACTCCCTCCCTCTCCCGAGAACTGCCCCGAGT<br>TCGCCCACGGGGGTTTACCGGGACTCGGGGCGGC<br>GGCGACTCCTCAGTCCCTAGACTAAGATGGCAGC<br>CGGTCCCGAGCCCCGACCACAGCTACTAGAGCGA<br>GTTAGCAGCTAAAGCGCAGGCGCCTGAGCGCGG<br>AAGTGGTCTAGACTACAAGCCCCAGCAGGCCTTG<br>CGCGCCAGCCGTCCGGCCCGCCCCTGGCTTGAAG<br>AGCTGGCTGCGGTGGTAAATCAGTTAAATCCCAG<br>TTTTCCTCAGCTCCAGGTGAGATGGGCGCGAGCG<br>GCTCCCAGCACCTCCAGGCTTTTTTTTTTTTTTAA<br>CACCCCGCCCACCAGAGCCTATAAGGACTGTGAT<br>TCTCAAACTATGGTGTGCATCAGAATCACCTAGAG<br>AGCTTATTAAAACTTACACTGCTGACCCCCACCCC<br>CAGAGTTTCTGATCAGAAGATCTGGCATAGGAGG<br>ATAGGGTCTGATAATTTGCATGTCCAACAAGCTGT<br>CTAGTGATACTGCTAGTGAGTGAAATCATAATTTG<br>AGAACCAATGGGCTATATAGCTTAGTTTATAACTTA<br>GTTGTTCTTTTTTCTTTTTCTTTTCCTTTTCTTTTTT<br>TTTCTTC
Mutant	hg38_dna<br>range=chr20:<br>34362774-34<br>363773	GGCCGCCGCCCCTCACAGGGCCACGTCGCCCGCG<br>CCGGCCCCGCCGCCGGGCCCCGCGCGTAGGCCGA<br>ACCGCTGGAGAGTGGCCCCAGGCGGGGCTGAGG<br>GGCGAGGGGCCGGACGGTACCCACGCCCGTCGT<br>GGCCCAGCCCTTTCACAACTTGGAGCCCGCGAGG<br>CGAAGGCCTCCGGAAGCCTGTGCCAGACACTGAT<br>CCCTCCGAACGTCCAGACAGCCCACCCCCACCGC<br>CCGGCCCGGGCCAGTAGCCCGAGCTCCCCGCGCC

	CCCGACAGACACCGGCTCTGGAAGAAGGAGCAG<br>CCGCGGCTCTGGGGTCCCGAACCGCGCGTCCCAC<br>TCCTCTACCCTTGTTCTCCCCGAACATTCTCATCC<br>GGACTCCCTCCCTCTCCCGAGAACTGCCCCGAGT<br>TCGCCCACGGGGGTTTACCGGGACTCGGGGCGGC<br>GGCGACTCCTCAGTCCCTAGACTAAGATGGCAGC<br>CGGTCCCGAGCCCCGACCACAGCTACTAGAGCGA<br>GTTAGCAGCTAAAGCGCAGGCGCCTGAGCGCGG<br>AAGTGGTCTAGACTGAGGTAAAAGTAGTTCCTTG<br>CGCGCCAGCCGTCCGGCCCGCCCCTGGCTTGAAG<br>AGCTGGCTGCGGTGGTAAATCAGTTAAATCCCAG<br>TTTTCCTCAGCTCCAGGTGAGATGGGCGCGAGCG<br>GCTCCCAGCACCTCCAGGCTTTTTTTTTTTTTAA<br>CACCCCGCCCACCAGAGCCTATAAGGACTGTGAT<br>TCTCAAACTATGGTGTGCATCAGAATCACCTAGAG<br>AGCTTATTAAAACTTACACTGCTGACCCCCACCCC<br>CAGAGTTTCTGATCAGAAGATCTGGCATAGGAGG<br>ATAGGGTCTGATAATTTGCATGTCCAACAAGCTGT<br>CTAGTGATACTGCTAGTGAGTGAAATCATAATTTG<br>AGAACCAATGGGCTATATAGCTTAGTTTATAACTTA<br>GTTGTTCTTTTTTCTTTTTCTTTTCCTTTTCTTTTTT<br>TTTCTTC
