# Supplementary figures and images for: Deletion of Wild-type p53 Facilitates Bone Metastatic Function by Blocking the AIP4 Mediated Ligand-Induced Degradation of CXCR4
Source: Front Pharmacol. 2022 Feb 1;12:792293. doi: 10.3389/fphar.2021.792293 (PMC8844016; doi:10.3389/fphar.2021.792293)

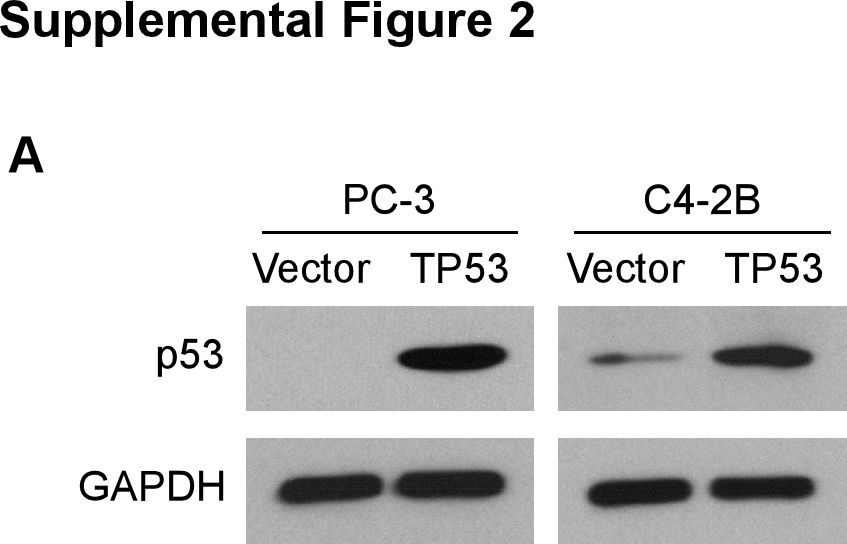

Supplement: Supplementary file 3 [file Image2.TIF]

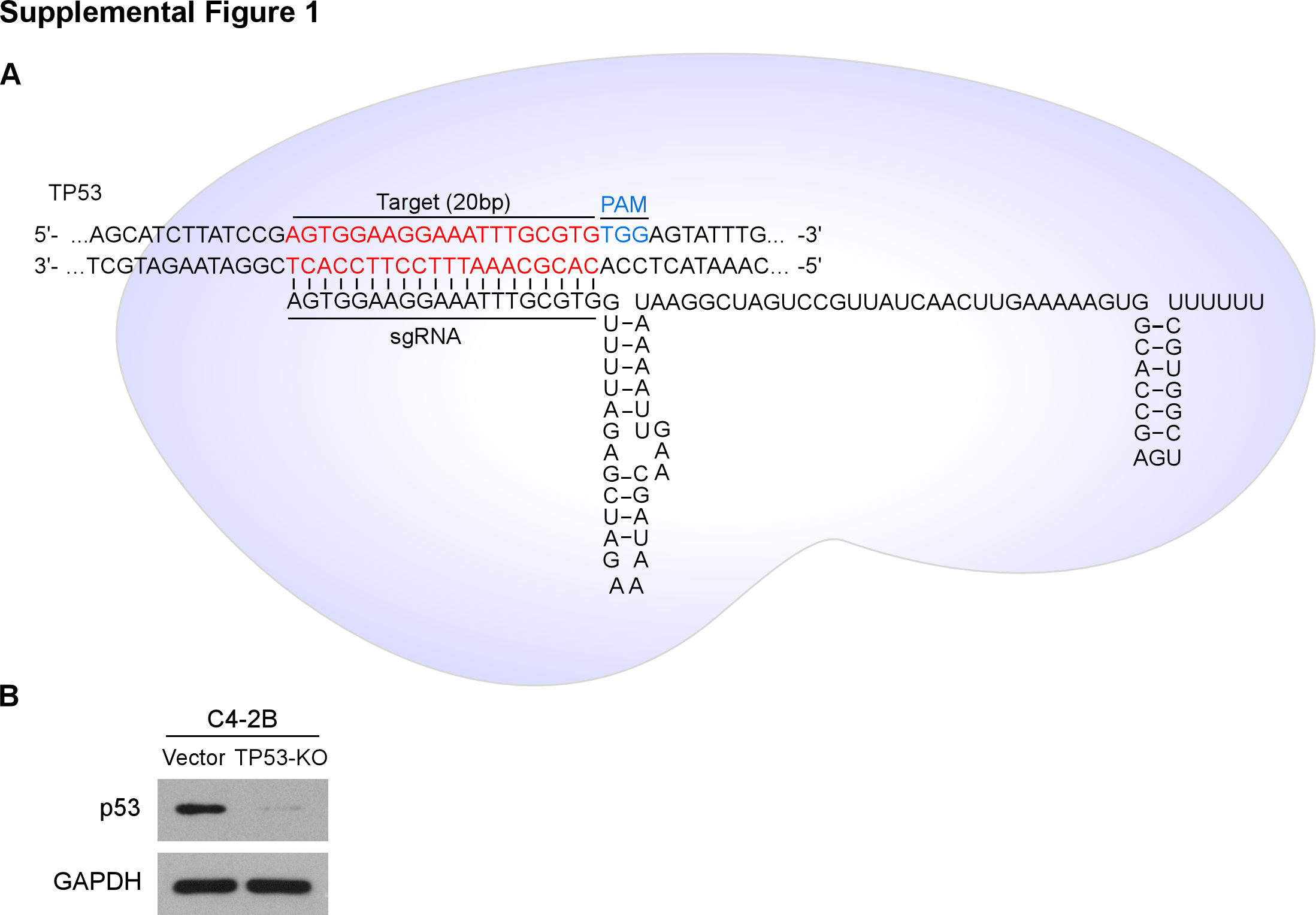

Supplement: Supplementary file 4 [file Image1.TIF]
